# Effects of Citric Acid and *Lactobacillus plantarum* on Silage Quality and Bacterial Diversity of King Grass Silage

**DOI:** 10.3389/fmicb.2021.631096

**Published:** 2021-02-26

**Authors:** Xuejuan Zi, Mao Li, Yeyuan Chen, Renlong Lv, Hanlin Zhou, Jun Tang

**Affiliations:** ^1^Key Laboratory of Ministry of Education for Genetics and Germplasm Innovation of Tropical Special Trees and Ornamental Plants, Key Laboratory of Germplasm Resources of Tropical Special Ornamental Plants of Hainan Province, College of Forestry, College of Tropical Crops, Hainan University, Danzhou, China; ^2^Tropical Crops Genetic Resources Institute, Chinese Academy of Tropical Agricultural Sciences, Danzhou, China

**Keywords:** king grass, *Lactobacillus plantarum*, citric acid, silage fermentation, bacterial community

## Abstract

To better understand the mechanism underlying the citric acid (CA)-regulated silage fermentation, we investigated the bacterial community and fermentation quality of king grass (KG) ensiled without (CK) or with *Lactobacillus plantarum* (L), CA and the combination of L and CA (CAL). The bacterial community was characterized by using the 16Sr DNA sequencing technology. The L and CA treatments altered the silage bacterial community of KG, showing reduced bacterial diversity, while the abundance of desirable genus *Lactobacillus* was increased, and the abundances of undesirable genus *Dysgonomonas* and *Pseudomonas* were decreased. The additives also significantly raised the lactic acid content, dropped the pH, and reduced the contents of acetic acid, propionic acid, and ammonia-N in ensiled KG (*P* < 0.01). Besides, the combination treatment was more effective on silage fermentation with the highest pH and lactic acid content, while the contents of acetic acid, propionic acid, and ammonia-N were the lowest (*P* < 0.01). Moreover, CAL treatment exerted a notable influence on the bacterial community, with the lowest operational taxonomic unit (OTU) number and highest abundance of *Lactobacillus.* Furthermore, the bacterial community was significantly correlated with fermentation characteristics. These results proved that L and CA enhanced the KG silage quality, and the combination had a beneficial synergistic effect.

## Introduction

As a *Pennisetum* grass species, king grass (*Pennisetum purpureum* Schumacher × *P. glaucum* (Linnaeus) R. Brown, KG) is widely distributed in the tropical and subtropical regions worldwide (Zhao et al., 2019). KG is a multifunctional plant, and it is extensively used in ecological environmental protection, bioenergy industry, and animal husbandry (Li et al., 2014; Li M. et al., 2019). KG vigorously grows in the summer or rainy season. However, its growth lags in the winter or drought season, resulting in biomass shortages (Li et al., 2014). Due to its seasonal harvest, KG should be properly preserved to provide a continuous supply. Ensiling is an approach for the long-term preservation of green forage. However, it is hard to make high-quality silage since KG or other *Pennisetum* grass has a higher lignin content and a lower content of soluble carbohydrates (Li et al., 2014; Desta et al., 2016).

The fermentation quality of silage mainly depends on the microbial community and its metabolites. Therefore, further study on the composition of silage microbial communities can provide a valuable scientific basis to enhance fermentation quality (Guo et al., 2018). Previous studies have investigated the silage microbial community composition of many forages, such as Alfalfa, corn, Napier grass, and so on (Guo et al., 2018; Xu et al., 2019; Yuan et al., 2019). Besides, the mechanism underlying the additive-improved fermentation quality has been analyzed, and various additives can enhance the silage fermentation quality in different ways (Guo et al., 2018; Xu et al., 2019; Yuan et al., 2019; Li F. et al., 2020).

Nevertheless, few reports have investigated the microbial composition of KG silage, and the mechanism underlying the response to additives remains unclear. Citric acid (CA) is a type of antioxidant, and it is also a safe additive used in the food industry (Li M. et al., 2020). Besides, CA can be used as a carbohydrate source to provide energy to lactic acid bacteria (LAB) and accelerate their growth (Li et al., 2016). Therefore, CA is considered as an ideal and novel silage additive to enhance the fermentation quality (Li et al., 2016; Ke et al., 2017, 2018; He et al., 2019b; Li M. et al., 2020; Lv et al., 2020). Our previous study has found that CA improves the silage quality though increasing the abundances of lactic acid-producing microorganisms, *Paenibacillus* and *Bacillus* (Li M. et al., 2020). Meanwhile, Lv et al. (2020) have reported that the addition of CA can increase the abundances of *Pediococcus* and *Lactobacillus*, while the abundances of *Enterobacter*, *Escherichia-Shigella*, and *Pantoea* are decreased, leading to enhanced *Amomum villosum* silage quality. However, the impacts of CA on the diversity of KG silage microorganisms remain largely unexplored.

In the present study, we hypothesized that LAB and CA had beneficial effects on the fermentation and microbes of KG silage, and their mixture might be shown a potential synergetic effect. Therefore, we aimed to assess the effects of LAB and CA on the microbial diversity and fermentation property of KG silage.

## Materials and Methods

### Silage Preparation

King grass (Reyan No. 4) was grown in the experimental base of the Chinese Academy of Tropical Agricultural Sciences (109°58′E, 19°52′N). The first cut KG of the vegetative stage (approximately 1.5–1.8 m height) was harvested and chopped into about 2-cm pieces by grass shredding machine (Donghong No. 1, Donghong Mechanical Equipment Co., Ltd., China). Four different treatments were conducted in our current study as follows: control (no additive, CK), *Lactobacillus plantarum* (L), CA, and the combination of *L. plantarum* and CA (CAL). According to the manufacturer’s guidelines, the additives were dissolved in sterile distilled water and then mixed thoroughly with the grass. An equal amount of sterile distilled water was added to the control group. Every treatment was carried out in triplicate. The application rate of *L. plantarum* (Snow Brand Seed Co., Ltd., Japan) and CA (Sinopharm Chemical Reagent Co., Ltd., China) was 1.0 × 10^5^ colony-forming units (cfu)/g of fresh matter (FM) and 5 g/kg of FM, respectively. Briefly, 200 g of KG was blended with additives, and the mixture was placed in plastic bags (35 cm × 12 cm × 5 cm; Guozhong Packing Co., Ltd., Haikou, China). A total of 60 bags (four treatments × five ensiling durations × three replicates) were prepared and stored at normal temperature (25–30°C). Three bags were used to determine the chemical composition and organic acid on days 1, 3, 7, 14, and 30. The microbial community was determined after 30 days of fermentation.

### Chemical Composition, Fermentation, and Microbial Analysis

Specimens were heated at 65°C for 72 h to determine the dry matter (DM) content, and dried materials were ground for chemical analysis. Crude protein (CP) and ether extract (EE) were determined according to the Guidelines of the Association of Official Analytical Chemists (AOAC 1990). Neutral detergent fiber (NDF) and acid detergent fiber (ADF) were determined as previously described by Van Soest et al. (1991). Water soluble carbohydrate (WSC) was determined according to a previously described method (Murphy, 1958). The fermentation quality of silage was determined using distilled water extracts. Briefly, 50 g wet silage was blended with 200 mL distilled water, followed by incubation at 4°C for 24 h and then filtration. The pH and concentrations of lactic acid, acetic acid, propionic acid, butyric acid, and ammonia-N were measured as previously established (Li et al., 2014). Microbial counts were analyzed using the plate count method on MRS agar, Violet Red Bile agar, and Rose Bengal agar as previously described (Li P. et al., 2019).

### Microbial Community Analysis

The above-mentioned extracts were used for the molecular analysis of the microbiota. Briefly, 20 mL filtrate was centrifuged at 12,000 *g*/min for 5 min, and the sediment was collected from the bottom. Microbial DNA was isolated from silage specimens with the E.Z.N.A^®^. soil DNA Kit (Omega Bio-Tek, Norcross, GA, United States) according to the manufacturer’s instructions. The concentration and purity of extracted DNA were assessed by NanoDrop 2000 UV-vis spectrophotometer (Thermo Fisher Scientific, Wilmington, DE, United States), and DNA integrity was confirmed by electrophoresis on 1% agarose gel. Primers 338F (5′-ACTCCTACGGGAGGCAGCAG-3′) and 806R (5′-GGACTACHVGGGTWTCTAAT-3′) were adopted to amplify the V3–V4 hypervariable regions of the bacterial 16S rRNA gene using a thermocycler PCR system (GeneAmp 9700, ABI, United States). After PCR products were purified and quantified, next-generation sequencing was carried out using Illumina MiSeq 2500 platform (Illumina, Inc., San Diego, CA, United States), and paired-end reads of 250 bp were generated.

The assembly of tags was carried out using filtered reads according to the principles as follows: overlap between paired-end reads should be more than 10 bp and less than 2% mismatch. The unique tags were obtained by removing redundant tags using software MOTHUR (Schloss et al., 2009). The abundance was then determined using the resultant unique tags. The high-quality reads were grouped into operational taxonomic units (OTUs) defined at a similarity of 97%. Diversity metrics were determined using the core-diversity plug-in within QIIME2^1^ (Callahan et al., 2016). The microbial diversity within an individual sample was assessed using the alpha diversity indices, including observed OTUs, Chao1 richness estimator, Shannon diversity index, and Faith’s phylogenetic diversity index. Beta diversity was analyzed to assess the structural variation of microbiota across specimens, and then principal component analysis (PCA) was conducted (Vázquez-Baeza et al., 2013). Appropriate methods were employed to identify the bacterial strains with different abundances among samples and groups (Segata et al., 2011). Unless otherwise specified above, parameters used in the analysis were set as default. The heat map function of the R software^2^ and genus information for the *Pennisetum sinese* silage were used to generate a heat map. The data were analyzed using the free online BMKCloud Platform.^3^ The sequencing data were submitted to the National Center for Biotechnology Information Sequence Read Archive database under the BioProject accession number of PRJNA556187.

### Statistical Analysis

The impacts of additives (*L. plantarum* and CA), ensiling duration, and their interactions were investigated by two-way analysis of variance using general linear model (GLM) procedure of SAS 9.3 software (SAS Institute Inc., Cary, NC, United States). Significant differences were compared using Duncan’s multiple range tests, and *P* < 0.05 was considered statistically significant.

## Results

### Chemical and Microbial Composition of Fresh KG

Table 1 shows the chemical and microbial compositions of fresh KG. The contents of DM, EE, CP, NDF, ADF, and WSC were 152.8, 60.2, 91.2, 765.3, 496.9, and 72.1 g/kg, respectively. Meanwhile, the number of LAB, yeast, and mold in fresh KG was 4.22, 2.78 and 3.04 cfu/g, respectively.

**TABLE 1:** Chemical and microbial composition of KG.

	DM	g/kg (DM)						Log_10_ cfu/g (FM)		
		OM	EE	WSC	CP	NDF	ADF	L	Yeast	Molds
King grass	152.8	898.4	60.2	72.1	91.2	765.3	496.9	4.22	2.78	3.04

*DM, dry matter; OM, organic matter; EE, ether extract; CP, crude protein; NDF, neutral detergent fiber; ADF, acid detergent fiber; L, lactic acid bacteria.*

### Chemical composition of KG during ensiling

Table 2 shows the chemical composition dynamics of KG silages during 30 days of ensiling, which was reduced when the ensiling duration was prolonged. The DM content of the CK group was the lowest compared with the other groups on day 30 (*P* < 0.05). The CP content in the CK and L groups was lower compared with the CA and CAL groups on days 14 and 30 (*P* < 0.05). The contents of NDF and ADF showed a similar reducing trend, although it was not significantly different from the CK group. Meanwhile, lower contents of NDF and ADF were found in the CA and CAL groups on day 30 (*P* < 0.05). Additionally, the ensiling duration (D) and additive treatment (T) remarkably affected the contents of DM, NDF, and ADF (*P* < 0.01), and the CP content was only influenced by D (*P* < 0.01). Besides, we also found a significant interaction between D and T for the contents of DM, NDF, and ADF (*P* < 0.01).

**TABLE 2:** Chemical composition of KG silage.

Item	Treatment	Ensiling days					SEM	*P*-value		
		1	3	7	14	30		D	T	D*T
Dry matter (g/kg FW)	CK	158.6a	145.1b	134.6c	129.7cdB	124.8dB	2.31	<0.01	<0.01	<0.01
	L	160.2a	155.0b	147.3c	140.8dA	138.7dA				
	CA	156.8a	144.5b	140.8b	137.6bcA	135.1cA				
	CAL	159.3a	156.9a	146.1b	142.7bcA	138.8cA				
Crude protein (g/kg DM)	CK	91.4a	88.3a	86.8ab	83.4bB	82.5bB	0.79	<0.01	0.62	0.05
	L	93.7a	90.5a	87.4ab	84.6abB	82.3bB				
	CA	93.1a	92.1a	91.5a	90.8abA	88.9abA				
	CAL	92.5a	91.9a	91.3a	90.5abA	88.6abA				
Neutral detergent fiber (g/kg DM)	CK	767.7	762.0	759.9A	754.1A	750.7A	10.32	<0.01	<0.01	<0.01
	L	771.3a	750.3ab	730.8bAB	720.3bB	701.5bB				
	CA	767.5a	749.85ab	714.9bB	661.4bC	633.8bC				
	CAL	778.2a	738.12ab	705.4bB	656.8bC	634.3bC				
Acid detergent Fiber (g/kg DM)	CK	490.4	488.2	481.7A	477.2A	474.4A	13.85	< 0.01	< 0.01	< 0.01
	L	497.6a	476.3a	450.0abB	436.4abB	421.6bB				
	CA	500.5a	488.6ab	442.9bB	393.5bB	357.7cB				
	CAL	489.4a	461.09ab	392.3bC	332.3cC	275.9dC				

CK, control; L, *Lactobacillus plantarum*; CA, Citric acid; CAL, Citric acid + *Lactobacillus plantarum*; FM, fresh matter; DM, dry matter; ND, not detected; “–”, default.D, ensiling days; T, treatment; D*T, interaction of ensiling days and treatment; SEM, standard error of means;Means with different letters in the same row (a–d) or column (A–D) differ (*P* < 0.05).

### Fermentation Property of KG During Ensiling

Table 3 illustrates the fermentation characteristic dynamics of KG silage. The pH in all groups was dramatically reduced (*P* < 0.05) during the 7 days of fermentation, while the lowest pH was found on day 30. Meanwhile, the highest and lowest pH on day 30 were found in the CK group and CAL group (*P* < 0.05), respectively. The lactic acid content in all groups was remarkably elevated during the fermentation, while the highest lactic acid content was found in the CAL group on day 30 (*P* < 0.05). The acetic acid content of the CA and CAL groups was lower compared with the CK and L groups on day 30 (*P* < 0.05). In the present study, the content of acetic acid in the CK and L groups was greater compared with the CA and CAL groups, which led to the pH raised in the CK and L groups accordingly. The propionic acid content in all groups was not significantly changed during ensiling, and the lowest content was found in the CAL group on day 30 (*P* < 0.05). Butyric acid was not detected in all groups, indicating that KG was well preserved. The ammonia-N content in all groups was dramatically increased (*P* < 0.05) after 3 or 7 days of fermentation, while the highest and lowest ammonia-N contents were found in the CK group and CAL groups (*P* < 0.05), respectively.

**TABLE 3:** Fermentation quality of ensiled KG.

Item	Treatment	Ensiling days					SEM	*P*-value		
		1	3	7	14	30		D	T	D*T
pH	CK	5.31a	4.91ab	4.78b	4.64bc	4.51cA	0.07	<0.01	<0.01	<0.01
	L	5.24a	4.84ab	4.54b	4.36bc	4.29cB				
	CA	4.91a	4.68ab	4.53b	4.44b	4.23cB				
	CAL	4.90a	4.75ab	4.54b	4.25bc	3.95cC				
Lactic acid (g/kg DW)	CK	22.8c	24.7cB	31.1bB	33.5abB	36.5aB	2.28	<0.01	<0.01	<0.01
	L	24.2c	26.0bcB	29.0bB	32.8abB	36.9aB				
	CA	21.1c	24.7cB	28.4bcB	34.4bB	41.1aB				
	CAL	27.4d	30.7cdA	37.9cA	50.9bA	63.7aA				
Acetic acid (g/kg DW)	CK	5.7c	11.3bc	16.3bA	24.4abA	27.6aA	1.65	<0.01	<0.01	<0.01
	L	5.7c	9.5bc	13.6bAB	20.5abAB	26.5aA				
	CA	5.9c	8.9bc	11.9bAB	18.1abAB	22.4aB				
	CAL	6.0c	9.6bc	12.1bAB	18.8abAB	23.5aB				
Propionic acid (g/kg DW)	CK	0.93A	0.88A	1.02A	0.85A	0.84A	0.05	<0.01	<0.01	<0.01
	L	1.15A	1.07A	1.02A	1.13A	0.92A				
	CA	0.82A	0.89A	1.01A	0.96A	0.70A				
	CAL	0.58B	0.62B	0.54B	0.48B	0.45B				
Butyric acid (g/kg DW)	CK	ND	ND	ND	ND	ND	–	–	–	–
	L	ND	ND	ND	ND	ND				
	CA	ND	ND	ND	ND	ND				
	CAL	ND	ND	ND	ND	ND				
Ammonia-N (g/kg DW)	CK	24.2c	28.5bcA	33.3bA	41.8abA	44.6aA	1.66	<0.01	<0.01	<0.01
	L	20.2b	24.6bAB	30.2abAB	32.6abB	36.5aB				
	CA	18.6b	22.4abAB	25.4abAB	28.7aB	30.9aC				
	CAL	18.1b	20.8abAB	21.5abB	22.6aBC	23.2aD				

CK, control; L, *Lactobacillus plantarum*; CA, Citric acid; CAL, Citric acid + *Lactobacillus plantarum*; DM, dry matter; ND, not detected; “–”, default. D, ensiling days; T, treatment; D*T, interaction of ensiling day and treatment; SEM, standard error of means; Means with different letters in the same row (a-d) or column (A-D) differ (*P* < 0.05).

### Microbiota Community of KG Silage

A total of 958,785 raw reads and 910,947 raw tags were generated, after an average of 70, 971 clean tags and 68,123 effective tags was obtained in each silage sample.

**FIGURE 1:**
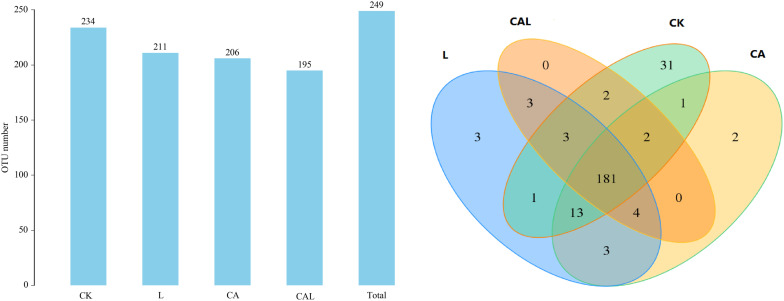
Venn analysis of OTUs for KG silage (CK, control; L, *Lactobacillus plantarum*; CA, Citric acid; CAL, Citric acid + *Lactobacillus plantarum*).

**FIGURE 2:**
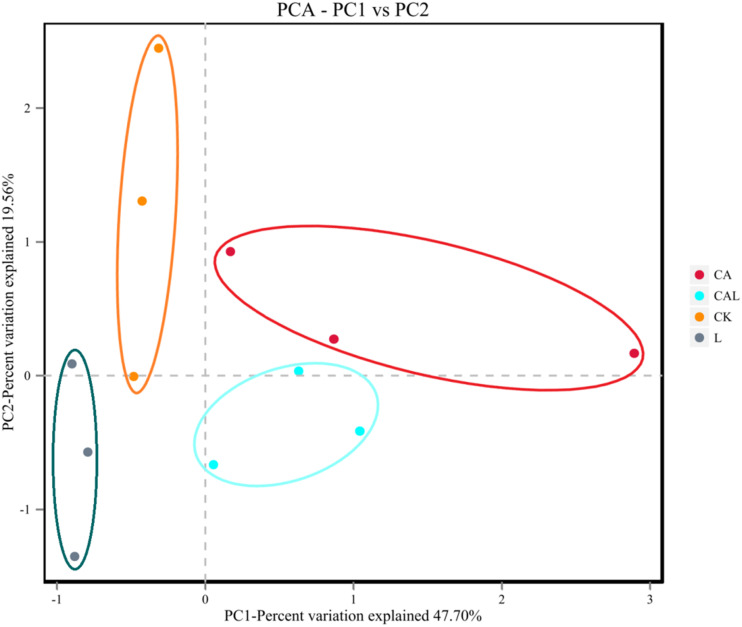
Beta-diversity of the bacterial community of KG silage (CK, control; L, *Lactobacillus plantarum*; CA, Citric acid; CAL, Citric acid + *Lactobacillus plantarum*).

Table 4 and Figure 1 show the α-diversity of the bacterial community of silages. Additive treatment affected the Ace, Chao 1, Shannon, and Simpson indices of bacterial diversity (Table 4). For community richness comparison, the Shannon index was lower, and the Simpson index was higher in the additive-treated groups compared with the CK group. A total of 249 OTUs were detected, with the highest number of OTUs found in the CK group (234), and the lowest number found in the CAL group (195) (Figure 1). Venn analysis exhibited that the additive treatment resulted in 181 common OTUs, and there were 31, 3, and 2 special OTUs in the CK, CA, and L groups, respectively. The PCA was employed to examine the correlations among the community structures of the silage bacterial community. A clear separation and difference of bacterial communities were found in four groups (Figure 2), suggesting that the bacterial composition was changed with different additive treatments. Therefore, we drew a conclusion based on the α-diversity and β-diversity that the LAB and CA treatments could affect the bacterial diversity and community structure of KG silage.

**TABLE 4:** Alpha-diversity of bacterial diversity of KG silage.

	OTU	Chao1	ACE	Shannon	Simpson	Coverage
CK	234	202.52	197.86	2.57	0.17	0.99
L	211	196.71	197.34	2.46	0.19	0.99
CA	206	186.24	178.33	2.16	0.19	0.99
CAL	195	195.78	194.08	2.06	0.26	0.99

*CK, control; L, *Lactobacillus plantarum*; CA, Citric acid; CAL, Citric acid + *Lactobacillus plantarum.**

**FIGURE 3:**
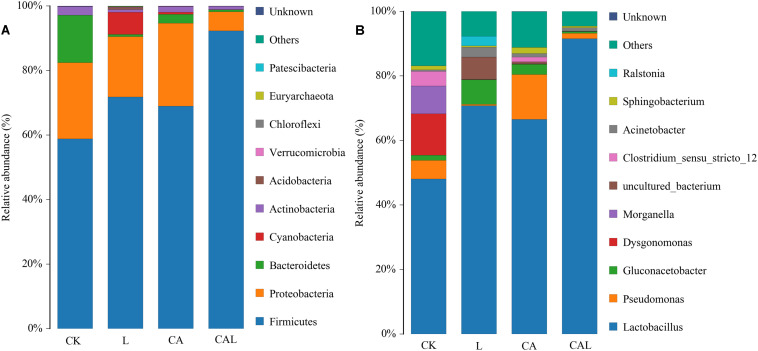
The bacterial community and abundance at the phylum **(A)** and genus **(B)** levels in KG silage (CK, control; L, *Lactobacillus plantarum*; CA, Citric acid; CAL, Citric acid + *Lactobacillus plantarum*).

Figure 3A describes the bacterial community at the phylum level. *Firmicutes* and *Proteobacteria* were dominant in all groups, *Bacteroidetes* was sub-dominant in the CK and CA groups, and *Cyanobacteria* was sub-dominant in the L group. The silage bacterial community was shifted upon the additives, the abundance of *Firmicutes* in additive treatment groups was increased, while the abundances of *Proteobacteria* and *Bacteroidetes* were decreased (except for the CA group) compared with the CK group. To further investigate the effects of additives on the bacterial community during ensiling, we examined the bacterial structures of KG silage at the genus level (Figure 3 B). *Lactobacillus* was predominant in the four groups. The sub-dominant microbes, in turn, were *Dysgonomonas*, *Morganella*, and *Pseudomonas* in the CK group, *Gluconacetobacter* in the L group, *Pseudomonas* and *Gluconacetobacter* in the CA group, and *Pseudomonas* in the CAL group. The abundance of *Lactobacillus* was increased along with the additive treatments, while the abundances of *Dysgonomonas* and *Pseudomonas* were decreased (except for the CA group). Particularly, CA treatment promoted the abundance of *Lactobacillus*, especially for the combination group, which accounted for as high as 95% of the total population.

**FIGURE 4:**
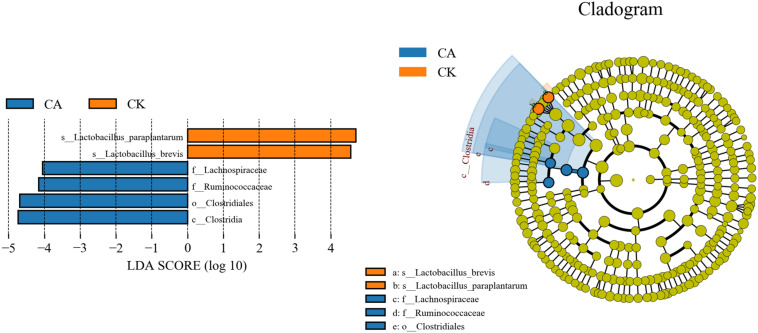
Comparison of microbial variations using the LEfSe online tool for KG silage (CK, control; CA, Citric acid).

The linear discriminant analysis (LDA) effect size (LEfSe) method was used to assess the differences in microbial community between four groups and explore the specific bacterial in each group (LDA score >4.0). Figure 4 shows that CA exerted a dramatic impact on the microbial community. *Lactobacillus paraplantarum* and *Lactobacillus brevis* were the most abundant species in the CK group, and *Lachnospiraceae* and *Ruminococcaceae* were the most abundant families in the CA group, which could be the biomarkers of different treatments.

**FIGURE 5:**
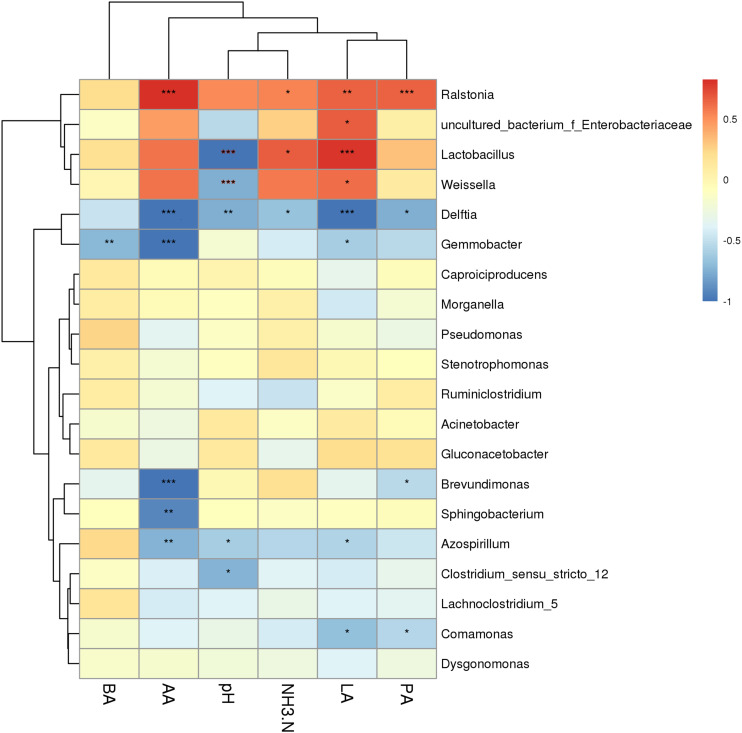
Correlation of the bacterial community and silage fermentation (CK, control; L, *Lactobacillus plantarum*; CA, Citric acid; CAL, Citric acid + *Lactobacillus plantarum*; LA, Lactic acid; AA, Acetic acid; PA, Propionic acid; BA, Butyric acid; NH_3_-N, Ammonia-N). *P < 0.05, **P < 0.01 and ***P < 0.001.

To better understand the silage fermentation process, we also assessed the correlation between the fermentation characteristics and the bacterial community of KG silage (Figure 5). The lactic acid was positively correlated with genera *lactobacilli*, *Weissella*, and *Enterobacter* (*P* < 0.05), while it was negatively correlated with genera *Delftia*, *Gemmobacter*, *Azospirillum*, and *Comamonas* (*P* < 0.05). The acetic acid was positively associated with genera *Ralstonia* (*P* < 0.001), while it was negatively associated with genera *Delftia*, *Gemmobacter*, *Brevundimonas*, *Sphingobacterium*, and *Azospirillum* (*P* < 0.01). The propanoic acid was highly and positively correlated with genera *Ralstonia* (*P* < 0.001), but negatively associated with genera *Delftia, Brevundimonas*, and *Comamonas* (*P* < 0.05). The pH was negatively correlated with genera *Lactobacillus*, *Weissella*, *Delftia*, *Azospirillum*, and *Clostridium* (*P* < 0.05). Moreover, the ammonia-N content was positively correlated with genera *Lactobacillus* and *Ralstonia* (*P* < 0.05), while it was negatively associated with genus *Delftia* (*P* < 0.05).

## Discussion

Compared with the previous reports, the DM of KG was lower, while the contents of EE, CP, NDF, and ADF were higher (Li et al., 2014; Li M. et al., 2019; Xu et al., 2019). Recently, Li D. et al. (2019); Li P. et al. (2019) have found that the contents of DM and CP are 239 and 133 g/kg in KG, respectively, which are much higher compared with the above-mentioned studies. The differences in forage quality may be caused by cultivation, climatic conditions, soil fertility, growth period, and harvest time. The WSC content is a key factor affecting the fermentation quality. The WSC content in KG was 72.1 g/kg, which met the minimum requirement (60–80 g/kg DM) for well-conserved silage (Smith, 1962). However, the tropical forages normally have coarse and stemmy structures and high fiber fraction, which can cause the bad quality of silage (Yu et al., 2011; Li et al., 2017). Some previous studies have confirmed that KG ensiled alone can not achieve high fermentation quality (Li et al., 2014; Li M. et al., 2019). The well-preserved silage needs an LAB number of more than 5.0 log_10_ cfu/g during ensiling (Cai et al., 1998). The lower LAB number and higher yeast and mold contents indicated that more LAB was required for preparing KG silage. Moreover, the undesirable yeast and mold could be inhibited by the low pH, leading to the wide application of organic acid in forage silage (Guo et al., 2018; He et al., 2019b). Therefore, it might be helpful to achieve higher silage quality by rapidly reducing pH and promoting fermentation through adding CA and *L. plantarum*.

In the present study, the CA and CAL groups contained higher CP content. This finding might be explained as the effect of CA, which could inhibit protein hydrolysis (Ke et al., 2018). The CA treatment dramatically reduced the contents of NDF and ADF in KG. However, the effect of CA on ADF and NDF was contrary to the report of Ke et al. (2018) in alfalfa silage. The divergence might be related to the different types of forage that KG in this study was Gramineae plant, while the alfalfa was Leguminosae plant, which contained a higher amount of structural fibers. This probably impacted the effect of CA on ADF and NDF during ensiling. Meanwhile, the positive effect of CA on forage chemical composition has been also reported in previous studies (Li et al., 2016; Ke et al., 2017, 2018; He et al., 2019b). These results suggested that a combination of *L. plantarum* and CA could improve the feeding value of KG to some extent.

Silage pH plays an important role in the evaluation of fermentation property, and silage with pH 4.2 or lower would be considered as well-fermented silage (Edwards and McDonald, 1978). In this study, the pH value of the additive-treated groups was close to or below 4.2, which could ensure good preservation of KG silage. Li et al. (2016) and He et al. (2019b) have shown a similar pH of CA-treated silage. In contrast, Ke et al. (2017, 2018) have shown a higher pH value in CA-treated alfalfa silage, which is even higher than 5.0, and such discrepancy may be attributed to the different application rates. The lactic acid in silage is the dominant fermentation product, which is another important evaluation index of silage quality. The addition of CA directly decreased the pH, which could inhibit harmful microorganisms and promote the reproduction and growth of LAB, leading to increased lactic acid content. Such finding was consistent with the previous data, indicating that CA can increase the lactic acid content, while different supplementation levels have various effects (Ke et al., 2017; He et al., 2019b). However, some studies have demonstrated the different effect. Li et al. (2016) and He et al. (2019b) have reported that CA treatment reduces lactic acid content in alfalfa and *Neolamarckia cadamba* leaf silage. Zhao et al. (2004) and Seo et al. (2007) have shown that released CA can be used by some yeast strains as a carbon and energy sources, and the growth of yeasts may be more competitive than LAB when an appropriate level of CA is applied, resulting in the lower lactic acid content. Therefore, the optimal amount of CA in different silage materials needs to be further studied. As the main metabolite of *Acetobacter* fermentation in silage, acetic acid is also a crucial index for the evaluation of silage quality. The higher acetic acid concentration always leads to a higher pH, which benefits the undesirable microorganism *Clostridia*, leading to reduced silage quality (Zheng et al., 2017). The ammonia-N content in silage often reflects the breakdown efficiency of protein to peptide. The comparatively low ammonia-N content in additive-treated silage could be attributed to the effect of lower pH values, indicating that the activity of protease was inhibited and preservation of more nutrients. The above-mentioned results indicated that the addition of *L. plantarum* and CA in the ensiling process could promote the fermentation quality, and the combination treatment more efficiently enhanced the fermentation. He et al. (2019b) have reported the profitable associated effects of *L. plantarum* and CA combination treatment on alfalfa silage. Similarly, Guo et al. (2018) have also found that the combination of hexanoic acid and *L.plantarum* can improve the fermentation characteristics compared with the single treatment in Napier grass silage.

In our study, the CK group showed the high Shannon, Ace, and Chao1 indices, as well as the low Simpson index mean high bacterial diversity. These results implied that additive treatment could reduce bacterial diversity. It could be explained that the LAB and CA treatment decreased the pH, inhibited the growth of harmful microorganisms, and promoted the growth of LAB species. When LAB became the dominant species, the bacterial diversity was decreased. Similar data have been reported by Ni et al. (2017) and (Wang et al., 2019a,b) in soybean, *Moringa oleifera* leaves and *Morus alba* leaves. Meanwhile, PCA indicated that a clear separation and difference of bacterial communities were found in different groups of KG silage, suggesting that the bacterial composition was changed in the ensiling process with different additive treatments. This could be explained by that CAs could provide additional substrates to LAB and accelerate its growth, while the addition of *L. plantarum* also increased desirable microorganisms.

In the present study, *Firmicutes* and *Proteobacteria* were the top two dominant phyla in KG silage, which was similar to previous studies (Xu et al., 2017; Dong et al., 2019). However, the total abundances of the two phyla were increased from approximately 80% to more than 95% after fermentation (Liu et al., 2019; Wang et al., 2019b). Such elevation might be attributed to different silage materials and treatments. Normally, as the major bacterial strain with desirable functions, the *Lactobacillus* is dominant in well-preserved forage silage, because it is responsible for driving lactic fermentation during ensiling (Bao et al., 2016; Ni et al., 2017, 2018; Guan et al., 2018; Liu et al., 2019). CA treatment also promoted the abundance of *Lactobacillus*, especially for the combination group, which accounted for as high as 95% of the total population. It could be attributed to that the addition of CAs could provide substrates and supplement energy to *Lactobacillus*, which was beneficial for its propagation. Besides, the acidic environment formed by the addition of CA also inhibited the undesirable microbes, which might be conducive to the growth of *Lactobacillus*. Similar data have also been found by Lv et al. (2020) that the addition of CA can increase *Pediococcus* and *Lactobacillus*, resulting in enhanced silage quality *of Amomum villosum*. However, our previous study has found that CA improves the silage quality through raising the abundances of *Paenibacillus* and *Bacillus*, and other studies have also shown that *Lactobacillus* is not the dominant bacterial strain in organic acid-treated silage (He et al., 2019a, 2020; Li M. et al., 2020). Therefore, the type of organic acids and the proportion of additives may affect the bacterial community. In contrast, *Dysgonomonas* is a facultative anaerobe, *Pseudomonas* is an aerobic bacterium, and both of them are undesirable microorganisms and can reduce the fermentation quality (Hofstad et al., 2000; Dunière et al., 2013). *Dysgonomonas* is rarely reported in silage, and its effect and mechanism in silage fermentation need to be further studied. *Pseudomonas* spp. is considered an undesirable bacterial strain for silage, because it may be associated with the production of biogenic amines, leading to decreased protein content and nutritional value (Dunière et al., 2013). In the present work, the LAB additive treatment markedly reduced the abundance of *Pseudomonas*, which was harmful to the silage quality and nutrient preservation. Similar findings have been reported in corn stover, alfalfa, *M. oleifera* leaves, and red clover (Xu et al., 2017; Ogunade et al., 2018; Dong et al., 2019; Wang et al., 2019b). *Gluconacetobacter* is widely used in the production of vinegar and wine, which is a type of acetic acid-producing bacterial strain, and can consume ethanol and sugar substances (Du et al., 2018; Huang et al., 2018). The higher abundance of *Gluconacetobacter* resulted in more acetic acid, leading to raised pH and impaired fermentation quality.

Silage fermentation is a very complex biological process involving a variety of microorganisms, and such a process produces many different metabolites during ensiling. *Lactobacillus* is the core microorganism during the ensiling process, *Weissella* is also widely distributed in silage, and both of them produce lactic acid and determine the silage quality (Cai et al., 1998). Similar to our findings, previous studies have reported that the concentration of lactic acid is positively correlated with genera *Lactobacillus* and *Weissella* in silage (Xu et al., 2017; Guan et al., 2018; Ogunade et al., 2018; Yang et al., 2019). Meanwhile, *Enterobacter* converts lactic acid to acetic acid and other organic acids, so it is positively correlated with lactic acid (Ostling and Lindgren, 2010). The ammonia-N is created by the synthetic effect of plant enzymes and microbial activity, and the reduction of silage ammonia-N was attributed to the rapid acidification of LAB. Therefore, *Lactobacillus* was sensitive to lower pH, contributing to the high correlation coefficients between ammonia-N and *Lactobacillus*. Our results were consistent with the other studies (Ogunade et al., 2018; Ren et al., 2019).

## Conclusion

The addition of LAB or CA significantly raised lactic acid content, decreased pH, and reduced contents of acetic acid, propionic acid, and ammonia-N in ensiled KG. The LAB and CA treatment also altered the bacterial community of KG silage, which reduced bacterial diversity. However, such treatments increased the abundance of desirable *Lactobacillus*, but decreased the abundance of undesirable *Dysgonomonas* and *Pseudomonas*. Besides, their combination treatment displayed a beneficial synergistic impact on silage fermentation, and notable influence on the bacterial community. Furthermore, the bacterial community was significantly correlated with fermentation characteristics. Collectively, our results confirmed that LAB, CA, and their combination exerted beneficial effects on KG silage fermentation.

## Data Availability Statement

The datasets presented in this study can be found in online repositories. The names of the repository/repositories and accession number(s) can be found below: https://catalog.data.gov/dataset/sequence-read-archive-sra, PRJNA556187.

## Author Contributions

ML, XZ, YC, and RL did the experimental design work. ML, XZ, YC, RL, and JT conducted the experiments. ML, XZ, YC, RL, JT, and HZ analyzed the data. ML, XZ, and YC wrote the manuscript. All authors read and approved the manuscript.

## Conflict of Interest

The authors declare that the research was conducted in the absence of any commercial or financial relationships that could be construed as a potential conflict of interest.

## Footnotes

1 https://docs.qiime2.org/2019.1/

2 http://www.r-project.org/

3 www.biocloud.net
